# Distinct Pattern of Microgliosis in the Olfactory Bulb of Neurodegenerative Proteinopathies

**DOI:** 10.1155/2017/3851262

**Published:** 2017-03-19

**Authors:** Zacharias Kohl, Johannes C. M. Schlachetzki, Judith Feldewerth, Philipp Hornauer, Martina Münch, Anthony Adame, Markus J. Riemenschneider, Jürgen Winkler, Eliezer Masliah

**Affiliations:** ^1^Department of Molecular Neurology, Friedrich-Alexander-University Erlangen-Nürnberg, Schwabachanlage 6, 91054 Erlangen, Germany; ^2^Department of Cellular and Molecular Medicine, University of California, La Jolla, San Diego, CA, USA; ^3^Departments of Neurosciences and Pathology, University of California, La Jolla, San Diego, CA, USA; ^4^Department of Neuropathology, Regensburg University Hospital, 93053 Regensburg, Germany

## Abstract

The olfactory bulb (OB) shows early neuropathological hallmarks in numerous neurodegenerative diseases, for example, in Alzheimer's disease (AD) and Parkinson's disease (PD). The glomerular and granular cell layer of the OB is characterized by preserved cellular plasticity in the adult brain. In turn, alterations of this cellular plasticity are related to neuroinflammation such as microglia activation, implicated in the pathogenesis of AD and PD, as well as frontotemporal lobe degeneration (FTLD). To determine microglia proliferation and activation we analyzed ionized calcium binding adaptor molecule 1 (Iba1) expressing microglia in the glomerular and granular cell layer, and the olfactory tract of the OB from patients with AD, PD dementia/dementia with Lewy bodies (PDD/DLB), and FTLD compared to age-matched controls. The number of Iba1 and CD68 positive microglia associated with enlarged amoeboid microglia was increased particularly in AD, to a lesser extent in FTLD and PDD/DLB as well, while the proportion of proliferating microglia was not altered. In addition, cells expressing the immature neuronal marker polysialylated neural cell adhesion molecule (PSA-NCAM) were increased in the glomerular layer of PDD/DLB and FTLD cases only. These findings provide novel and detailed insights into differential levels of microglia activation in the OB of neurodegenerative diseases.

## 1. Introduction

Hyposmia is a frequent symptom in neurodegenerative diseases, such as Alzheimer's disease (AD) [1], and synucleinopathies, including Parkinson's disease (PD), Parkinson's disease dementia (PDD), and dementia with Lewy bodies (DLB) [2]. Moreover, hyposmia was observed in several other neurodegenerative disorders like frontotemporal lobe degeneration (FTLD), corticobasal degeneration, and Huntington's disease [3–5]. The early onset of olfactory dysfunction even prior to typical motor or cognitive symptoms has received tremendous attention for PD and AD, possibly implicating olfactory dysfunction as an early biomarker for the identification of patients-at-risk [6, 7]. Several postmortem studies identified the accumulation of typical protein deposits in the olfactory bulb (OB)/olfactory tract, namely, amyloid *β* (A*β*), hyperphosphorylated Tau (p-Tau) within neurofibrillary tangles (NFT), and alpha-synuclein (a-Syn) aggregates in the form of Lewy bodies (LB) and Lewy neurites [8]. Importantly, the prototypical neuropathology occurs in the OB early in the disease course and even prior to characteristic clinical signs. The olfactory system is involved in early neuritic Braak stages of AD [8, 9], and the earliest neuropathological stage of PD (Braak LB stage 1) is characterized by a-Syn accumulation in the anterior olfactory nucleus of the OB [10]. Moreover, Lewy pathology is present in the glomerular layer and the granular cell layer in most symptomatic and asymptomatic cases of different Lewy body diseases [11]. In contrast, the occurrence of TAR DNA-binding protein 43 (TDP43) pathology in the OB of FTLD and related disorders is far less well described [12, 13].

Chronic neuroinflammation represents an important feature in the pathophysiology of various neurodegenerative disorders [14]. For instance, common genetic variants within the human leucocyte antigen (HLA) region show increased risk for AD [15], PD [16], and FTLD [17], indicative for a possible contribution of the immune system to the pathogenesis of these disorders. Microglia activation is well described in AD [18], and the expression of microglial markers in various brain regions, including brain stem, striatum, and cortex, is increased in PD as well [19]. The accumulation of p-Tau and a-Syn in AD and PD, respectively, is accompanied with an increased density of microglia in the anterior olfactory nucleus of the human OB [20]. Upon pathological stimuli, microglial cells transform from a ramified morphology into amoeboid-shaped cells [21, 22]. Moreover, an increased number of activated microglia was also described in frontal and temporal grey and white matter regions of FTLD [23].

Interestingly, the OB is a region of the adult mammalian brain where newly generated neurons migrate from the subventricular zone via the rostral migratory stream to the OB and integrate into existing neuronal networks in two neurogenic niches of the OB, the glomerular, and the granular cell layer [24]. The glomerular layer consists of synapses between the terminals of the olfactory nerve and the dendrites of mitral, periglomerular, and tufted cells, as well as a heterogeneous population of juxtaglomerular neurons; in contrast, the granular cell layer is densely composed of GABA-ergic inhibitory neurons [25]. Adult OB neurogenesis was predominantly characterized in rodents, as well as in adult primates [26]. Whether OB neurogenesis occurs in adult humans is still under debate for several years [27–29]. Nevertheless, immature neurons expressing polysialylated neural cell adhesion molecule (PSA-NCAM) and, to a much lesser amount, neuroblasts expressing doublecortin (DCX) were observed in the OB of healthy adults [29]. While the analysis of OB neurogenesis in several animal models of PD or AD show reduced survival of newly generated OB neurons [30, 31], a detailed analysis of neurogenic markers in these disorders is lacking. Interestingly, altered activity of microglia may substantially contribute to changes of cell proliferation in neurogenic areas of the adult human brain, for example, the OB [32]. A recent study described alterations of cell proliferation in the hippocampus of human PD cases, mainly due to increased microglial proliferation [33]. Thus, the present analysis focused on the number of microglia cells in the glomerular and granular cell layer, compared to the olfactory tract, further determining whether specific disease pathologies lead to a distinct pattern of microglia activation in these regions. In addition, we used the marker cluster of differentiation (CD) 68 to further identified activated microglia and quantified CD 68+ cells in the OB of the respective disease entities. Moreover, we addressed the question whether the level of microglia proliferation is altered in the OB from patients with AD, PDD/DLB, or FTLD. Finally, we addressed the question whether alterations in microglial activity also affects the expression of immature neuronal markers in the OB.

## 2. Materials and Methods

### 2.1. Postmortem Brain Tissue

Human postmortem olfactory bulb tissue was obtained from the Alzheimer Disease Research Centre (ADRC) at the University of California, San Diego (UCSD). In most cases, patients received neuropsychological testing at UCSD ADRC as part of a structured annual examination; Blessed Information-Memory-Concentration (BIMC) and Minimental State Examination (MMSE) scores are reported (Table 1). Subjects included in the present study had cognitive testing performed within 12 months prior to death. Institutional board review was obtained from the UCSD Human Research Protections Program in accordance with the Health Insurance Portability and Accountability Act. Written informed consent was obtained from all patients or their guardians.

For analysis, 10 clinical diagnosed and neuropathologically confirmed AD patients, six patients with diagnosed DLB or PDD, and eight FTLD patients were included in the study. Moreover, six healthy subjects as controls without neurological or psychiatric diseases and without AD or a-Syn pathology were included. The clinicopathological data of all subjects is summarized in Table 1. The diagnosis was based on standardized neuropathological examination of different brain areas independently by two experienced neuropathologists.

### 2.2. Tissue Processing

OBs were bilaterally dissected at autopsy and fixed in 4% phosphate buffered formaldehyde solution for a period of approximately 4–8 weeks. Tissue was dehydrated in graded ethanol and embedded in paraffin. From the paraffin blocks 6 *μ*m horizontal sections were cut on a rotating microtome and mounted on positively charged glass slides (Superfrost Plus; Thermo Fisher scientific, Waltham, MA, USA). Sections were dried in a stove overnight before further processing.

### 2.3. Immunohistochemistry

For immunohistochemistry, sections were heated in a stove at 65°C for 30 minutes, prior to being deparaffinized in Histoclear (National Diagnostics, Atlanta, GA, USA) and rehydrated by a graded series of ethanol (100%, 95%, 70%, 50%, and 20%) and PBS containing 0.5% Tween20 (Sigma, St. Louis, MO, USA) (PBS-T). For antigen retrieval, different protocols were evaluated; for the present study, tissue was pretreated in 98°C citrate buffer solution (10 mM, pH 6.0) for 15 min in the microwave. After pretreatment the sections were allowed to regain room temperature (RT) and rinsed for 30 min in 50% methanol/PBS containing 0.6% H_2_O_2_ to block endogenous peroxidase activity. Next, nonspecific binding sites were blocked with 3% serum from the respective species of the primary antibody in PBS-T for 60 min. Tissue on the slides was outlined with a DAKO pen (DAKO, Carpinteria, CA, USA) and incubated with primary antibody diluted in blocking solution overnight in a humified chamber at 4°C. Primary antibodies used were as follows: PSA-NCAM (1 : 200; AB5324, Millipore), ionized calcium binding adaptor molecule 1 (Iba1; 1 : 200, Wako, Richmond, VA, USA), CD68 (PGM1; 1 : 200; #M0876, Dako, Glostrup, Denmark), p-Tau (1 : 200; gift from Dr. Peter Davies), a-Syn (1 : 50; clone 15G7, Enzo Life Science, Famingdale, USA), tyrosine-hydroxylase (TH; 1 : 250; Millipore, Billerica, MA, USA), TDP43 (1 : 300; clone 2E2-D3, Abnova, Taipei City, Taiwan), and DCX (1 : 200, Santa Cruz Biotechnology, Santa Cruz, CA, USA). The following day sections were extensively washed in PBS and incubated for 1 h in the biotinylated secondary antibody (donkey-derived; 1 : 100 in blocking serum; Jackson) followed by HRP-labeled avidin biotin complex (1 : 400; ABC, Vectastain ABC Elite Kit; Vector laboratories, Burlingame, CA, USA) for 45 min at room temperature (RT). Staining was finally visualized using 3,3-diaminobenzidine (DAB; Vector laboratories) as chromogen, and counterstain was performed with a diluted hematoxylin solution for 1 min. After dehydration in graded ethanol series, the sections were cleared in Histoclear and coverslipped with Entellan (Merck, Darmstadt, Germany). In order to visualize glomerular layer, granular cell layer, and olfactory tract of the OB, sections from each case were visualized using Nissl-staining, dehydrated, and coverslipped accordingly.

### 2.4. MCM2 Immunohistochemistry

The marker minichromosome maintenance protein 2 (MCM2) has been used in cancer research as a marker to identify dividing cells in situ and is able to detect cells throughout the cell cycle starting in early G1 phase. Moreover, MCM2 was also used to study cellular plasticity and neurogenesis in human brain tissue [34]. For the current study we modified a protocol described previously [34]. Sections were deparaffinized and rehydrated as described above. For subsequent antigen retrieval slides were placed in citrate buffer (pH 6.0) and heated for 35 min at 99° in a household pressure cooker device, followed by a blocking step in 3% donkey serum in TBS-T (TBS, 0.1% Triton-X) at pH 7.4 for 1 h. Afterwards sections were exposed to the primary antibody against MCM2 (1 : 200; goat polyclonal; Santa Cruz, Santa Cruz, CA, USA) in blocking solution (TBS, 0.1% Triton-X, 3% donkey serum) overnight at 4°C. On the following day, after several washing steps in TBS-T the sections were incubated in the secondary biotinylated antibody (1 : 500; rabbit polyclonal; Vector Laboratories, Burlingame, CA, USA) for 1 h at RT. To increase binding, primary and secondary antibody steps were repeated for 1 h at RT each. Then, sections were preincubated in 1% BSA in TBS for 30 min at RT to increase signal amplification. To visualize the signal, sections were treated with avidin-biotin complex (ABC, Vectastain ABC Elite Kit, Vector Laboratories, Burlingame, CA, USA) in 1% BSA and TBS for 1 h at RT. Sections were incubated with biotin tyramide (1 : 50; Perkin Elmer) in TBS with 0.03% H_2_O_2_ for 10 min at RT for signal amplification and again incubated in ABC as above for 45 min at RT. Finally, signal was visualized with DAB as described above, including counterstaining, dehydration steps, and mounting.

### 2.5. Immunofluorescence

To determine the number of proliferating microglia in the OB we performed triple fluorescence staining labelling for Iba1 and MCM2, including DAPI as nuclear counterstain. Therefore, we adapted the protocol for MCM2 from above and extended the blocking step to 3 h in 3% donkey serum (in TBS-T). The dilutions of the primary antibodies were adapted (rb-anti-Iba1 1 : 800, gt-anti-MCM2 1 : 500), as well as the concentration of secondary antibodies used. To label Iba1+ cells, a donkey anti-rb Alexa Fluor 568 antibody was applied. To further increase specificity of the MCM2 staining, a biotinylated donkey anti-goat antibody (1 : 200; Jackson Immuno Res., West Grove, USA) was used. The signal for MCM2 was amplified by using biotin tyramide (see above) and further incubated with Alexa 488 labeled streptavidin (1 : 800; Life technologies, Carlsbad, CA, USA) for 30 min. To reduce background fluorescence staining, we additionally applied a 1% Sudan Black B solution (Sigma, St. Louis, USA) dissolved in 70% ethanol for 3 minutes. Finally, DAPI (1 : 1000; Sigma) was used as nuclear counterstain; sections were coverslipped using Prolong Gold (Life technologies, Carlsbad, CA, USA).

### 2.6. Semiquantitative Analysis

OB sections were selected and analyzed where both the glomerular layer and the granular cell layer were fully preserved and clearly identifiable, and the olfactory tract was present. The numbers of PSA-NCAM+, MCM2+, and Iba1+ cells were quantified by using a Zeiss AxioImagerM2 microscope (Carl Zeiss, Göttingen, Germany) with a CCD color video camera (QImaging, Surrey, BC, Canada) and a LUDL MAC6000 motorized stage with StereoInvestigator software version 10 (MicroBrightfield Inc., Colchester, VT, USA). To prevent experimenter bias, all OB sections were coded. The glomerular layer, granular cell layer, and olfactory tract were identified within the StereoInvestigator software using a 20x objective resulting in a counting frame of 500 × 350 *μ*m. Five randomly chosen and representative areas of the glomerular layer, granular cell layer, or olfactory tract were analyzed through the whole thickness of the section from each case, determining the mean number of cells expressing the respective marker.

For quantification of CD68+ cells in all three regions, a 40x objective was used for proper identification of labelled cells. Within the StereoInvestigator software, a counting frame of 250 × 175 *μ*m was determined, 5 randomly chosen representative areas of all three regions of interest were counted, and the mean number of CD68+ cells was calculated. Due to unspecific signal, one sample from the AD group was excluded from this analysis.

While microglia with small cell body diameters and ramified processes may rather reflect a resting state, activated amoeboid microglia show larger cell bodies. Accordingly, microglial cell bodies were quantified by measuring the diameter of Iba1+ cells [35, 36]. Using the measure tool within the StereoInvestigator software, ten representative cell bodies in each counting frame were measured and the mean diameter was determined for each case for all three regions. We further determined the amount of proliferating microglia and quantified the percentage of Iba1+ microglia that coexpress MCM2 using the identical Zeiss AxioImager M2 microscope connected with an additional AxioCam MRm camera (Zeiss). Two sections of each case were used to identify 50 Iba1+ cells that were further analyzed for colabelling with MCM2. To exclude unspecific staining, we only rated MCM2+ signals if a typical nuclear expression pattern matched the respective DAPI signal.

### 2.7. Statistical Analysis

The mean number and size of labelled cells in the OB were calculated for each region and disease entity. One-way ANOVA was performed followed by Kruskal Wallis post hoc test if significant differences were detected; *p* < 0.05 was assumed to be significant. Statistical analysis was performed using Prism 5 (Graph Pad Software Inc., La Jolla, CA, USA).

## 3. Results

### 3.1. Specific Neuropathological Features in the Glomerular Layer, Granular Cell Layer, and Olfactory Tract of the Human OB in AD, PDD/DLB, and FTLD

**Figure 1:**
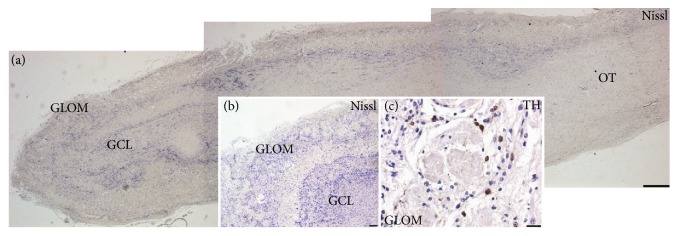
Overview of the human olfactory bulb (OB) analyzed in the study: (a) Nissl staining of the human olfactory bulb showing the glomerular layer (GLOM), the granular cell layer (GCL), and the olfactory tract (OT). Inserts: (b) higher magnification of the most rostral portion of an OB depicting the distribution of Nissl stained cells in the GLOM and the GCL. (c) The GLOM was further identified by staining for tyrosine hydroxylase (TH) expressing neurons within glomeruli, counterstained with hematoxylin. OB tissue from control case, scale bars represent 500 *μ*m in (a) and 50 *μ*m in inserts (b, c).

**Figure 2:**
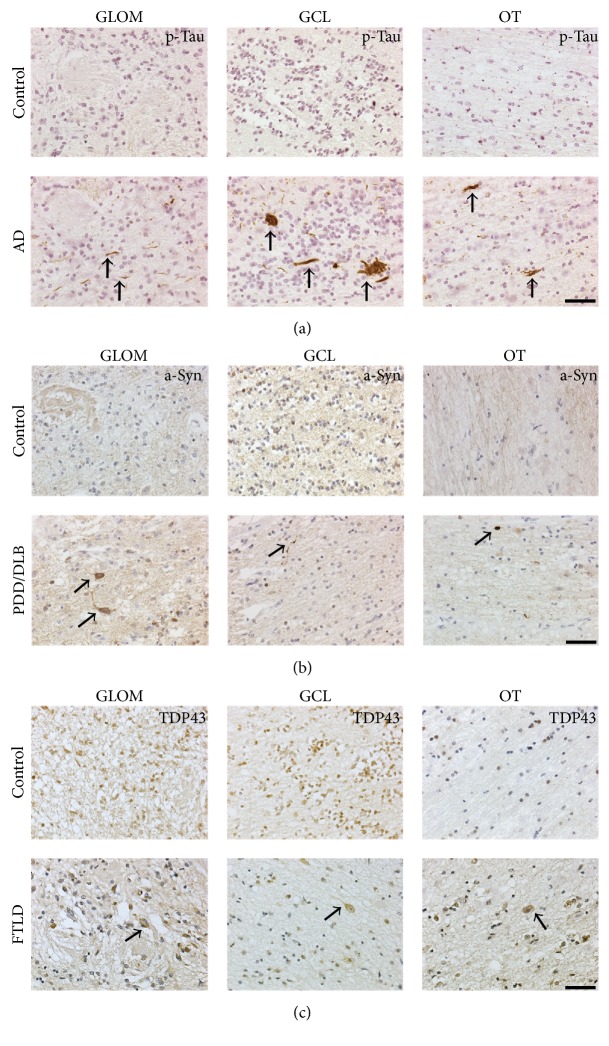
Typical neuropathological hallmarks of Alzheimer's disease (AD), Parkinson disease dementia/Lewy body disease (PDD/DLB), and frontotemporal lobe degeneration (FTLD) in the GLOM, the GCL, and the OT compared to controls. (a) Aggregates of hyperphosphorylated Tau (p-Tau; brown) were present in all regions analyzed in AD cases. (b) Accumulation of alpha-synuclein (a-Syn; brown) was detected in the GLOM, the GCL, and the OT of PDD/DLB cases, but not in controls. (c) While controls showed a physiological nuclear staining for unphosphorylated TDP43 in all regions investigated, a shift of TDP43 signal (brown) from the nucleus to the cytoplasm, together with a loss of nuclear staining, was observed in the GLOM, GCL, and OT in FTLD. Counterstain with diluted hematoxylin or hemalaun (blue). Scale bars represent 50 *μ*m.

First, we determined the regions of interest for all cases using Nissl stainings (Figures 1(a) and 1(b)). The preserved shape of the GLOM was confirmed by labelling dopaminergic interneurons expressing TH [37] (Figure 1(c)). Moreover, we specifically excluded the anterior olfactory nucleus within the olfactory tract from our analysis. Further, the presence of disease specific proteins was determined in the glomerular layer, granular cell layer, and olfactory tract of AD, PDD/DLB, and FTLD patients. Aggregated p-Tau immunoreactivity was present in the three regions of AD cases (Figure 2(a)), but also to a lesser extent in PDD/DLB and FTLD confirming previous findings [20]. Aggregated a-Syn was detected in cells of the glomerular layer, granular cell layer, and olfactory tract in PDD/DLB cases (Figure 2(b)). Less than 30% of AD and FTLD patients presented with a-Syn accumulation. A typical neuropathological hallmark of FTLD is the shift of unphosphorylated TDP43 protein from the nucleus to the cytoplasm, leading even to a reduction or loss of nuclear staining. We detected this altered staining pattern in the glomerular layer, granular cell layer, and olfactory tract in some, but not in all FTLD cases (5/8) (Figure 2(c)), while a nuclear staining pattern was present in all control cases.

### 3.2. Increased Microgliosis in the OB of AD, PDD/DLB, and FTLD

**Figure 3:**
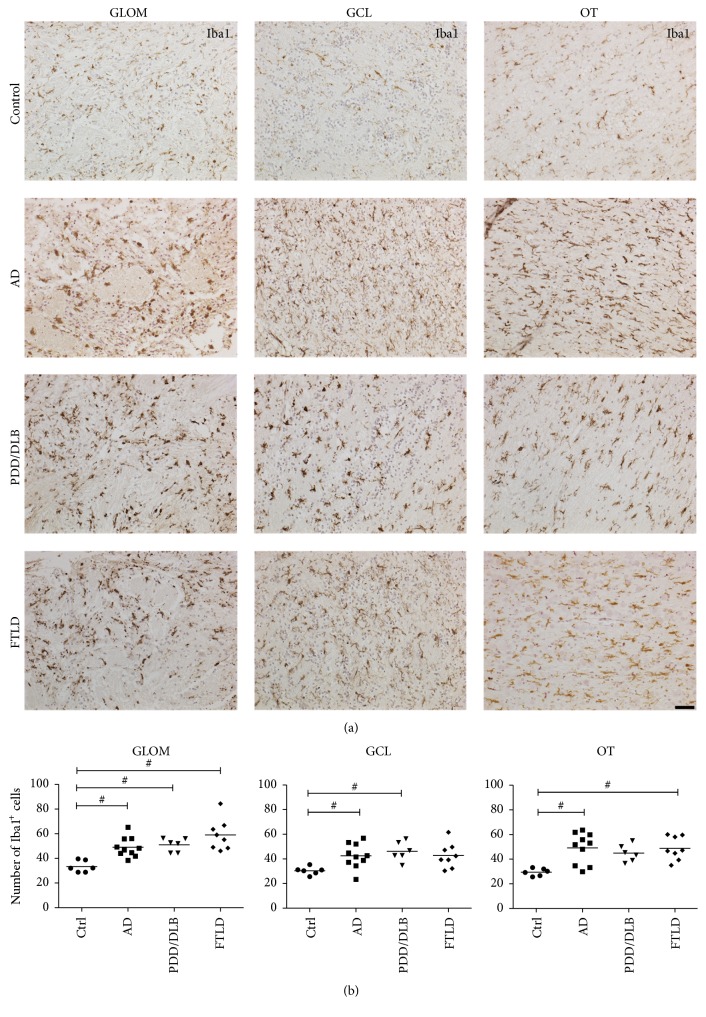
Increased numbers of Iba1 expressing microglia cells in the GLOM, GCL, and OT of the OB from AD, PDD/DLB, and FTLD cases. In the GLOM a significant increase in the number of Iba1+ microglia compared to controls was present in these diseases. Interestingly, the number of microglia was significantly higher in the GCL in AD and PDD/DLB patients, while the number of Iba1+ microglia was increased in the OT of AD and FTLD patients, but not PDD/DLB cases compared to controls. Representative images showing microglia in respective regions of the OB in (a) and quantification of Iba1+ cells in (b). 1-way ANOVA, Kruskal Wallis post hoc test, ^#^*p* < 0.05. Scale bar represents 50 *μ*m.

Previous studies have described increased proliferation of microglia in the hippocampus and the anterior olfactory nucleus of the OB in AD and PD patients [20, 33]. We focused our analysis on the number of Iba1 expressing microglia in the glomerular layer, granular cell layer, and olfactory tract of controls and AD, PDD/DLB, and FTLD cases. Iba1 is expressed by all subtypes of microglia, and the staining nicely labels cytoplasm as well as processes, allowing a detailed morphological characterization (Figure 3(a)). Compared to controls, we detected a significant increase in the number of Iba1 positive microglia in the glomerular layer for all diseases (Figure 3(b)). Moreover, 40% and 52% more Iba1 expressing cells were observed in the granular cell layer of AD and PDD/DLB cases, respectively. In the olfactory tract, the number of Iba1 positive cells was significantly higher in AD and FTLD only. Taken together, these findings implicate that the neurodegenerative proteinopathies examined result in an increased number of Iba1+ microglia in the human OB for all regions analyzed.

**Figure 4:**
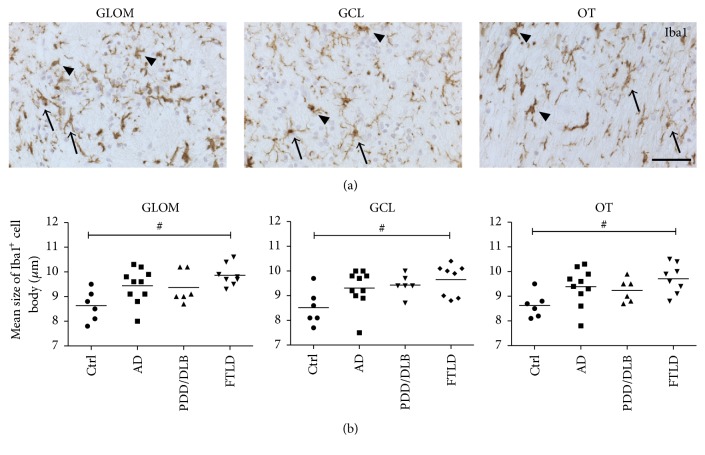
Analysis of the size of Iba1+ cell somata in the GLOM, GCL, and OT of the OB in respective neurodegenerative diseases. (a) Example images of Iba1+ microglia in all three regions of the OB, showing resting Iba1+ cells with smaller cell somata and long processes (arrows), compared to activated Iba1+ microglia with an increased cell soma size and short or no processes (arrowheads). (b) While the mean diameter (*μ*m) of somata of Iba1+ microglial cells was not significantly increased in all regions of AD and PDD/DLB cases, cell somata were significantly larger in FTLD compared to controls, suggesting an activation of Iba1+ microglia in FTLD. 1-way ANOVA, Kruskal Wallis post hoc test, ^#^*p* < 0.05. Scale bar represents 50 *μ*m.

We further aimed to distinguish resting microglia with smaller cell bodies and multiple ramified processes from activated amoeboid microglia presenting larger cell bodies with few short processes [35]. By comparing the mean diameter of Iba1+ cells in the glomerular layer, granular cell layer, and olfactory tract between controls, AD, PDD/DLB, and FTLD cases, we observed an increased diameter of Iba1+ cells in all three regions analyzed, however only reaching significance for the FTLD cases (Figures 4(a) and 4(b)). Taken together, pathological proteinopathies lead to increased numbers of Iba1 expressing microglia in neurogenic and nonneurogenic regions of the OB. Particularly, microglia appear to be more activated in FTLD cases, as shown by a significant increase in cell diameter representing an amoeboid phenotype.

### 3.3. Microglia Activation in the OB of AD, PDD/DLB, and FTLD Determined by CD68 Expression

**Figure 5:**
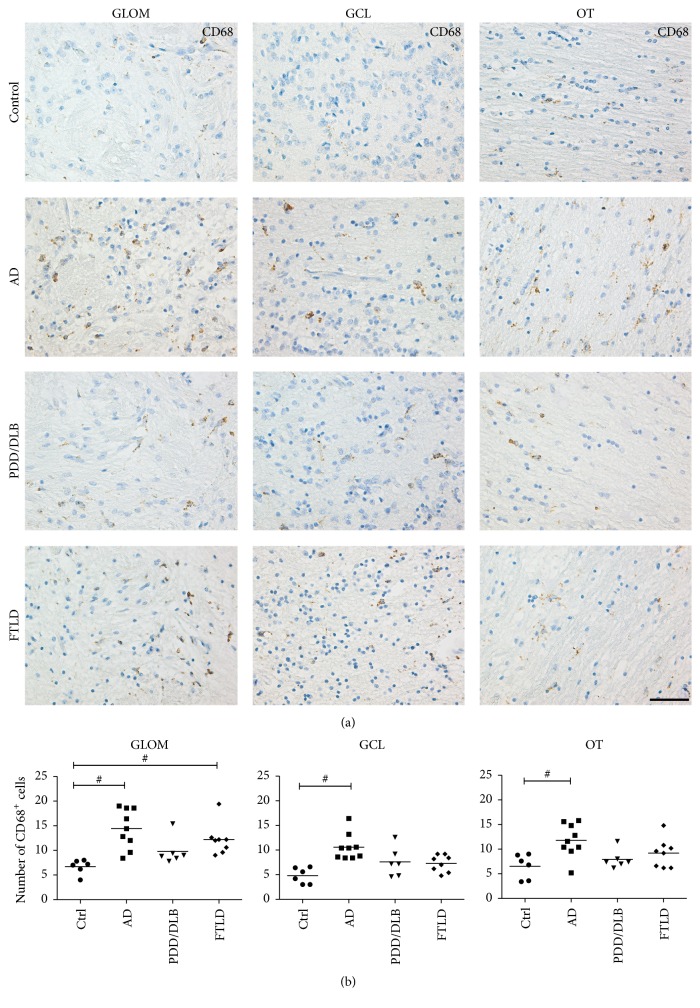
Increased numbers of microglia expressing CD68 in the GLOM, GCL, and OT of the OB from AD and FTLD cases. In the GLOM, CD68+ cell numbers were significantly increased in AD and FTLD compared to controls. Moreover, the number of CD68+ expressing cells in the GCL and OT was significantly higher in AD only. (a) Representative images showing CD68+ cells in respective regions of the OB and quantification of CD68+ cells in (b). 1-way ANOVA, Kruskal Wallis post hoc test, ^#^*p* < 0.05. Scale bar represents 50 *μ*m.

To further delineate the level of microglial activation we analyzed the expression of CD68, labelling lysosomal glycoproteins in microglia and indicative for phagocytic activity [38], in the glomerular layer, granular cell layer, and olfactory tract of AD, PDD/DLB, FTLD, and control cases (Figure 5(a)). The quantification revealed a strong increase of CD68+ microglia in all three regions of the OB from AD samples (Figure 5(b)). Moreover, we also detected microglia activation in FTLD in the glomerular layer, while in PDD/DLB the number of CD68+ was not altered in the three regions analyzed. These findings underline the notion that microglia activation in the OB represents an important feature in neurodegenerative diseases, predominantly in AD.

### 3.4. Proliferating Iba1+ Microglia in the OB from AD, PDD/DLB, and FTLD Patients

**Figure 6:**
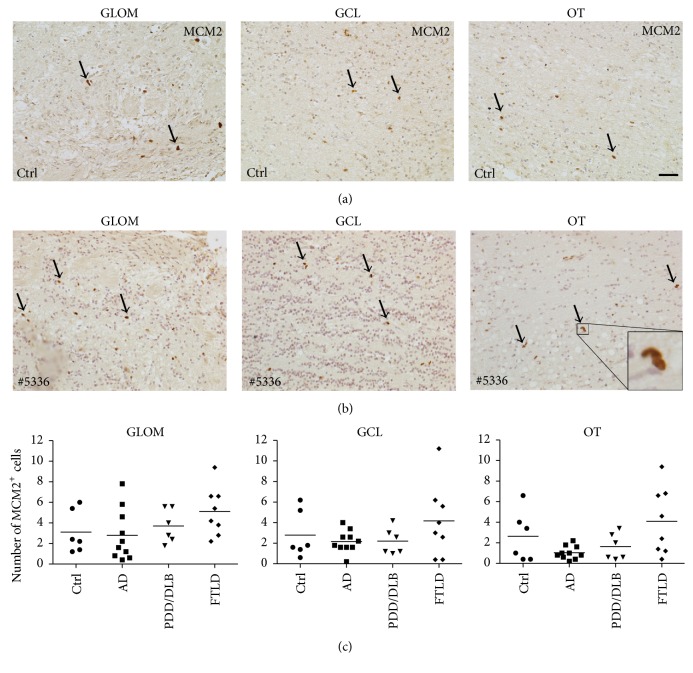
The number of proliferating cells in the GLOM, GCL, and OT of the OB was very low and not altered in AD, PDD/DLB, or FTLD compared to controls. Representative images depicting proliferating cells expressing the marker MCM2 in the GLOM, GCL, and OT in a control (a) and in FTLD ((b) case #5336), with higher magnification of typical doublet of MCM2+ cells (insert). The quantification of MCM2-positive cells revealed no significant difference between controls and AD, PDD/DLB, or FTLD for GLOM and GCL, as well as for the OT (c). 1-way ANOVA, *p* > 0.05 for all groups. Scale bar represents 50 *μ*m.

Next, we compared the number of proliferating cells expressing the marker MCM2 in the glomerular layer, granular cell layer, and olfactory tract of control, as well as AD, PDD/DLB, or FTLD cases. We clearly identified MCM2 expressing cells in all three areas of the OB, displaying the typical morphology for proliferating cells, such as small size, condensed nuclei, and doublets of cells (Figures 6(a) and 5(a), higher magnification in insert). Nevertheless, the number of MCM2 expressing cells did not vary significantly between regions analyzed. Moreover, there was no significant difference between the numbers of MCM2 positive cells in all three regions of controls compared to AD, PDD/DLB, or FTLD samples (Figures 6(b) and 5(b)). In conclusion, the rate of cell proliferation in these regions of the OB was not altered between all groups.

**Figure 7:**
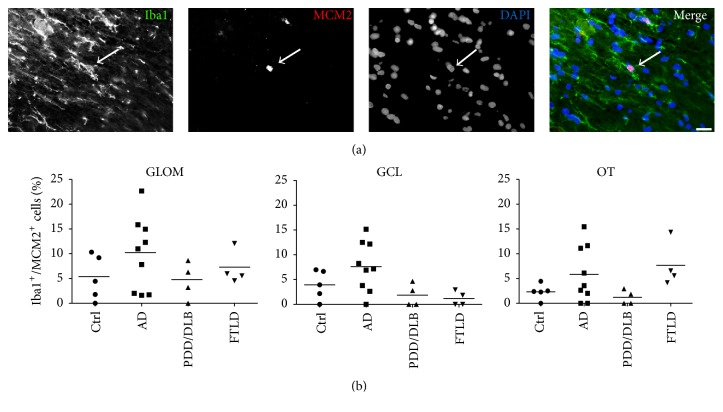
A small proportion of Iba1+ microglia was expressing MCM2, however without differences between controls and AD, PDD/DLB, or FTLD in the GLOM, GCL, and OT. (a) Triple-immunostaining showing a representative proliferating microglia cell expressing MCM2 and Iba1, DAPI as nuclear stain. (b) Percentage of Iba1+ microglia coexpressing the proliferation marker MCM2 in the three regions of the OB of controls and AD, PDD/DLB, and FTLD cases remains unaltered. 1-way ANOVA, *p* > 0.05 for all groups. Scale bar represents 20 *μ*m.

Then we focused our analysis of the three OB regions on the number of proliferating microglia and determined the percentage of Iba1+ cells coexpressing MCM2. Here, half of the AD cases presented a higher number of proliferating microglia in the glomerular layer, granular cell layer, and olfactory tract however without reaching significant difference compared to controls. The percentage of Iba1+/MCM2+ cells in PDD/DLB and FTLD cases remained unchanged (Figures 7(a) and 7(b) and Figures 6(a) and 6(b)). Due to structural limitations for immunofluorescent stainings on some samples, 8 cases (*n* = 1 from controls and AD group, *n* = 2 from PDD/DLB group, and *n* = 4 from FTLD group) were excluded from the analysis.

### 3.5. Expression of PSA-NCAM in the Glomerular Layer, Granular Cell Layer, and Olfactory Tract

**Figure 8:**
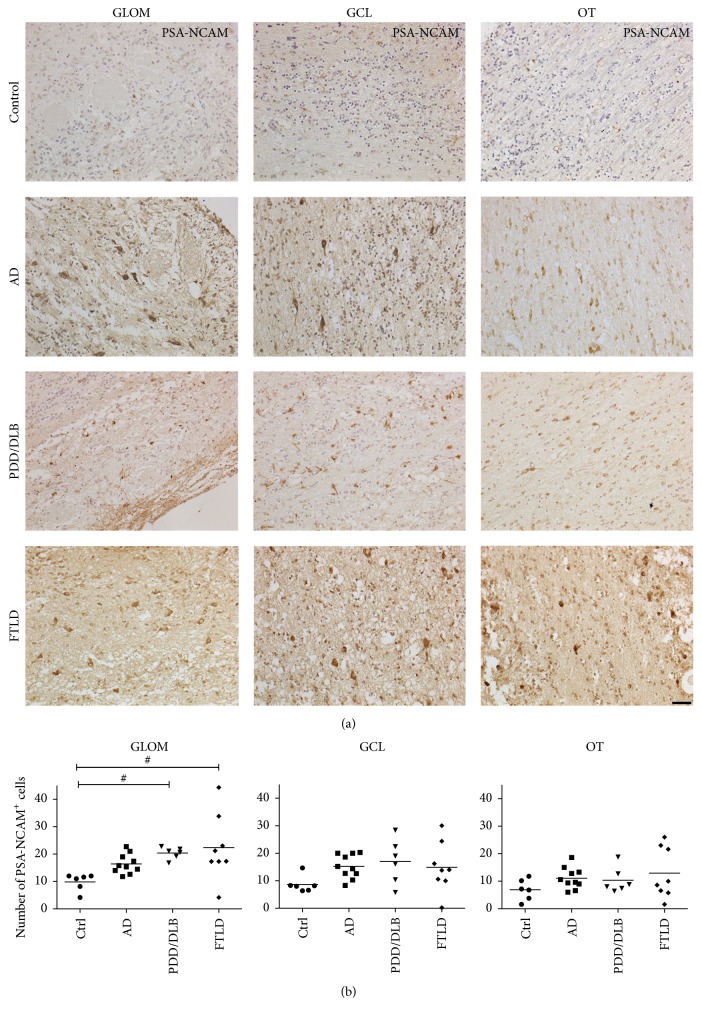
Increased number of cells expressing PSA-NCAM in the GLOM of PDD/DLB and FTLD patients. The number of cells expressing the immature neuronal marker PSA-NCAM was increased in the GLOM from PDD/DLB and FTLD cases, but not in AD patients, while in the GCL and the OT the number of cells was not altered compared to controls. (a) Representative images of cells expressing PSA-NCAM in all regions analyzed from controls, AD, PDD/DLB, and FTLD cases. Quantification presented in (b). 1-way ANOVA, Kruskal-Wallis post hoc test, ^#^*p* < 0.05. Scale bar represents 50 *μ*m.

We further determined the expression of markers for immature or developing neurons in neurogenic regions such as the glomerular and the granular cell layer of the OB of the different cohorts and therefore analyzed the distribution of cells expressing PSA-NCAM. We identified PSA-NCAM expressing cells with typical processes in the glomerular and granular cell layer of all cases, to a lesser extent in the olfactory tract as well. The quantification of PSA-NCAM expressing cells revealed an increased number in the glomerular layer of DLB/PDD as well as in FTLD cases (Figures 8(a) and 8(b) and Figures 7(a) and 7(b)). In contrast, the number of PSA-NCAM expressing cells in the glomerular layer of AD cases remained unaltered. Furthermore, the number of PSA-NCAM+ in the granular cell layer and the olfactory tract was not increased in AD, PDD/DLB, and FTLD compared to controls. In addition, we were not able to reliably detect cells expressing DCX in all areas examined.

## 4. Discussion

We described in detail distinct disease pathology, the expression of immature neural markers, and particularly the level of microglia activation in three different regions of the OB from patients with AD, PDD/DLB, and FTLD compared to controls. We detected an increased number and soma diameter of Iba1+ microglia both in the neurogenic regions of the OB, the glomerular and the granular cell layer, and in the nonneurogenic olfactory tract in all three proteinopathies. Moreover, CD68+ cells, also representing microglial activation, were increased particularly in AD and to a lesser extend in FTLD. Interestingly, the expression of the immature neuronal marker PSA-NCAM was significantly increased in the glomerular layer in PDD/DLB and FTLD only.

Olfaction is early affected in the course of these diseases, and the abundance of pathology in the OB reflects the severity of respective pathologies in other brain areas in these disease entities [8]. We consistently detected typical AD pathology, namely, aggregation of p-Tau in the three different areas of the OB analyzed, confirming previous observations of NFTs in the OB of AD patients [39], including the anterior olfactory nucleus [20], as well as the glomerular layer [12]. We further observed accumulation of a-Syn in the glomerular layer, granular cell layer, and olfactory tract of PDD/DLB cases, known to be present early in the disease course [10] and described in the anterior olfactory nucleus as well as the peripheral layers of the OB [12, 40]. Furthermore, by using an antibody against unphosphorylated TDP43, about 2/3 of the FTLD cases showed a typical cytoplasmic accumulation of TDP43 together with a loss of nuclear staining in all OB regions studied, representing the pathological relocalization of TDP43 [41]. This is in contrast to a previous study that failed to detect TDP43 pathology in the OB of FTLD cases despite the usage of the identical antibody [12]. Still, the significance of a TDP43 related neuropathological phenotype in the OB remains elusive, in particular as only about 30% of a larger cohort of ALS and ALS-FTLD cases presented with typical cytoplasmic TDP43 inclusions in the OB, irrespective of cognitive impairment [13].

Neuroinflammatory processes, including microglia activation, play an important role in dementia, particularly in AD (reviewed in [42]), PD (reviewed in [43]), and FTLD [23]. In addition, chronic anti-inflammatory treatment reduces the risk to develop PD [44], even leading to the development of clinical trials for neurodegenerative diseases using compounds inhibiting microglia activation [45]. A significant higher number of Iba1+ microglia was present in the glomerular layer, granular cell layer, and olfactory tract of AD brains compared to controls, emphasizing that neurogenic as well as nonneurogenic regions of the OB show microgliosis in response to AD pathology. This finding was further supported by the increase of CD68+ cells in all regions of AD cases, indicative of phagocytic activity of microglial cells. Furthermore, significant more Iba1+ expressing microglia were visible in the glomerular and granular cell layer from PDD/DLB patients as well. The microglial cells had larger cell somata representative of an amoeboid appearance and indicative for activation [36]. Upon exogenous activation, microglia extend its processes to the site of pathology, migrate to the lesion, and initiate an innate immune response, in particular via activation of toll-like receptors resulting in the production of proinflammatory cytokines like tumor necrosis factor-*α*, interleukin- (IL-) 1*β*, or IL-6. Overall, it is important to note that by using current markers it is not possible to clearly distinguish activated microglia from infiltrating macrophages, in particular in human brain samples [46, 47].

In AD, several lines of evidence show a direct response of microglia to soluble A*β* oligomers and A*β* fibrils [48]. Taken together, this leads to the hypothesis that the pathological accumulation of A*β* represents a key factor driving neuroinflammatory responses in AD [42, 49].

The early involvement of the OB in the neuropathology of synucleinopathies, in particular PD and DLB [10], as well as impaired olfactory function preceding the appearance of classical motor symptoms [50], raised considerable interest for neuroinflammatory processes in this region. Microglial activation was described in postmortem OB tissue of PD patients, as well as in the MPTP mouse model of PD, where early microgliosis was paralleled by increased IL-1 *α* and IL-1*β* levels in the OB [51]. A recent study described an increased number of activated microglia in the anterior olfactory nucleus of AD as well as PD cases, together with strong correlation with A*β* load in the AD brains [20]. In contrast, the amount of a-Syn pathology did not correlate with microglia activation, and no colocalization of A*β* or a-Syn aggregates with amoeboid microglia was present [20]. Interestingly, a recent study detected unaltered numbers of Iba1+ microglia in the frontal and temporal cortex of DLB patients [52]. Whether microglia activation in the OB is directly caused by the presence of A*β* or a-Syn, or a secondary inflammatory response to the disease process remains elusive. Additionally, it is not possible to directly link microglia activation to impaired olfaction in these neurodegenerative diseases.

In FTLD, findings about neuroinflammatory processes are still very limited: A pilot PET imaging study using the [^11^C](R)-PK11195 ligand identified microglia activation in frontotemporal brain regions of FTLD patients [53]. Just recently, a neuropathological investigation of 78 FTLD cases observed increased microgliosis in the cortical and subcortical grey matter of both frontal and temporal cortex [23]. Moreover, reduced function of the triggering receptor expressed on myeloid cells 2 (TREM2), preferentially expressed by microglia, was observed in AD, but also in FTLD cases [54]. We observed a significantly increased number of Iba1+ microglia both in the glomerular layer and in the olfactory tract of the OB from FTLD patients. Moreover, Iba1+ microglial cells showed significantly larger cell somata, suggestive of an activated state and implicating for the first time a significant involvement of the OB in FTLD. Finally, in the glomerular layer of the OB the number of activated microglia expressing CD68 was significantly increased, as already described in cortical brain regions [23], further supporting the notion of a possible role of inflammatory processes in the pathogenesis of FTLD.

We further addressed the question whether proliferation of microglial cells in the OB might also be altered in AD, PDD/DLB, or FTLD, possibly representing another mechanism of activation of the innate immune system [49]. In general, cell proliferation in all diseases, determined by the expression of MCM2 [33], was unaltered compared to controls. We furthermore defined the amount of proliferating microglia, represented by Iba1+ expressing cells colabeled with MCM2. Here, we detected a higher rate of proliferating microglia in AD cases, although without reaching statistical significance, while in PDD/DLB and FTLD an unchanged fraction of proliferating microglial cells was present. This is in accordance with recent data showing increased microglial proliferation in the temporal cortex of AD brains, mediated by the activity of the colony stimulating factor 1 receptor signaling pathway [55]. Interestingly, our findings in PDD/DLB relate well to data showing increased microglia proliferation in the hippocampus only in presymptomatic incidental Lewy body disease, but not in defined PD patients [33], similar to an increased toll-like receptor 2 expression in the hippocampus of incidental Lewy body disease only [56]. These findings may indicate that proliferation of microglia, in particular in synucleinopathies, might be an early event, possibly declining towards more advanced stages of the disease.

We also detected an increased number of cells expressing the immature neuronal marker PSA-NCAM in the glomerular layer of PDD/DLB and FTLD cases, though we were not able to detect DCX expressing cells in the OB regions analyzed. Still, as PSA-NCAM is expressed by mature interneurons in various brain regions and its expression is associated with features of plasticity and remodeling [57], we rather speculate that the increased expression of PSA-NCAM represents a response to altered microglial activity, but not newly generated neuroblasts. Moreover, the interaction of microglia with neurons may be influenced by PSA proteins via sialic acid-binding immunoglobulin-type lectin 11 (Siglec-11) [58]. In addition, a specific role of microglia activity for the generation of new neurons has been described in the prototypical neurogenic regions of rodents [32, 59, 60]. Physiologically, specialized microglial cells in the hippocampal dentate gyrus and in the subventricular zone support clearance of apoptotic new-born neuroblasts. In contrast, different detrimental challenges including neurodegenerative processes as well as aging trigger the production of proinflammatory cytokines by microglia leading to decreased neurogenesis [60]. Currently, evidence exists for the generation of new neurons in the adult human hippocampus, based on neuropathological analysis as well as ^14^C birth dating approaches [61, 62]. However, a limited number of studies addressed the impact of neurodegenerative processes like in AD or PD on hippocampal neurogenesis [63, 64]. In contrast, adult neurogenesis in the human subventricular zone/OB system, is, if at all, very limited. While PSA-NCAM expressing cells persist in the subventricular zone, the rostral migratory stream, and the OB in adult humans [29], the number of DCX+ neuroblasts in the subventricular zone and further migrating along the rostral migratory stream declines rapidly after birth; latest in early infancy, DCX+ neuroblasts are very rare in the rostral migratory stream and not present in the OB [28, 29]. A study using ^14^C birth dating method also concluded that there is little or no postnatal neurogenesis in the human OB [65]. In various animal models of AD and PD, an impaired generation of new OB neurons, particularly in the glomerular and granular cell layer, was detected [30, 66, 67]. In contrast, human postmortem studies showed conflicting results in regard of reduced cell proliferation in the subventricular zone of human PD patients [68, 69]. Additionally, no altered expression of proliferation or neurogenic markers were observed in the subventricular zone of AD patients [63].

## 5. Conclusion

Taken together, neurogenic and non-neurogenic regions of the human OB from AD, PDD/DLB, and FTLD patients contain PSA-NCAM expressing cells without further signs for the generation of new adult neurons. More importantly, microgliosis is present in response to proteinaceous aggregation, particularly in AD, resulting in increased microglial activation, determined by Iba1+ and CD68 analysis, but not an enhanced proliferation of microglia, specifying the impact of microglia activation in the OB of different neurodegenerative disorders.

## Figures and Tables

**Table 1:** Clinical and neuropathological characteristics of subjects included.

Group	Case #	Sex	Age	Brain weight	Disease duration (y)	MMSE	BIMC
Ctrl	5398	f	89	1202	—	30	m.d.
	5402	m	95	1302	—	30	m.d.
	5410	m	82	m.d.	—	m.d.	m.d.
	5455	f	93	1044	—	29	1
	5483	f	96	m.d.	—	29	0
	5495	m	86	m.d.	—	25	6

AD	5380	M	85	1284	7	24	7
	5384	F	97	860	18	m.d.	m.d.
	5391	M	91	1256	19	4	31
	5394	M	81	1002	8	m.d.	33
	5405	F	77	912	21	m.d.	m.d.
	5406	F	77	1232	m.d.	m.d.	33
	5419	F	46	644	10	12	28
	5440	F	73	820	12	m.d.	33
	5451	F	82	1044	18	1	29
	5457	M	77	1252	14	17	16

PDD/DLB	5377	M	87	1395	6	24	2
	5399	M	92	1240	m.d.	29	1
	5421	M	86	1360	7	27	2
	5462	M	81	1752	12	26	2
	5489	M	82	m.d.	10	24	3
	5499	M	76	m.d.	8	21	8

FTLD	5336	F	74	958	8	m.d.	33
	5395	F	82	900	5	6	m.d.
	5416	M	85	1202	15	7	30
	5424	M	72	910	16	m.d.	33
	5432	F	87	1084	10	1	27
	5448	M	87	1280	4	13	20
	5470	M	68	1426	m.d.	9	24
	5505	F	67	1310	15	8	27

Ctrl: nondemented controls, AD: Alzheimer's disease, PDD/DLB: Parkinson's disease dementia/dementia with Lewy bodies, FTLD: frontotemporal lobar degeneration, m.d.: missing data, MMSE: minimental status exam, and BIMC: Blessed Information Memory Concentration.
